# A powerful HUPAN on a pan-genome study: significance and perspectives

**DOI:** 10.20892/j.issn.2095-3941.2019.0317

**Published:** 2020-02-15

**Authors:** Yingyan Yu, Chaochun Wei

**Affiliations:** ^1^Department of Surgery, Ruijin Hospital, Shanghai Jiao Tong University School of Medicine, Shanghai Key Laboratory for Gastric Neoplasms, Shanghai 200025, China; ^2^School of Life Sciences and Biotechnology, Shanghai Jiao Tong University, Shanghai 200240, China

Recently, a report explaining the construction of a Human Pan-genome ANalysis (HUPAN) system attracted wide attention in the biomedicine community. The original article was published in *Genome Biology*, a leading international journal in genomics^1^. Many researchers, particularly those conducting basic medical research, were interested in knowing what type of technology HUPAN is and how it might affect the study of human diseases. Here, we provide a concise explanation of this analysis system and its biological importance, especially its potential effects on human disease research.

## Human genome project (HGP)

HGP is a large program in the life science field; it began in the United States (US) in the late 1980s and early 1990s^2^. In its initial stage, genome sequencing technology was not well developed and was relatively expensive. Shortly after the US government announced the launch of the HGP in 1993, the Chinese government announced that the National Natural Science Foundation of China would fund the HGP for Chinese scientists. China is a very large country that is home to one-fifth of the world’s population and 56 ethnic groups. If the international human genetic database lacks information on the Chinese genome, it would be incomplete^3^. In addition, scientists from France, Germany, the United Kingdom, and Japan were involved in the HGP study. After more than 10 years, through the efforts of scientists from 20 genome centers worldwide, a human reference genome was released^2^. On April 14 2003, the governments of the US, Britain, China, France, Germany, and Japan simultaneously announced that the expected sequencing work had been completed under the HGP and celebrated this great project carried out by life science researchers from 6 countries^4^.

Study of the human genome is highly dependent on the development of DNA sequencing technology and bioinformatics. Advances in DNA sequencing technology have enabled the genetic information in the cell nucleus to be presented in the real world. The development of bioinformatics has enabled analysis of massive amounts of genome sequencing data and has rapidly translated genome sequence information into new knowledge in biomedicine. Since its invention in 1977, DNA sequencing technology has undergone several iterations from first generation sequencing technology (Sanger sequencing), also known as chain termination DNA sequencing; to second-generation sequencing technology (a new generation of sequencing technology), including pyrophosphate sequencing^5^; to third-generation sequencing technology, including real-time single-molecule sequencing and Oxford nanopore sequencing. With the development of DNA sequencing technology, the throughput (the amount of sequencing data per single run) and lengths of sequencing reads are increasing.

## Conventional route of human genome study

Currently, the most commonly used sequencing method in human genome research is second-generation sequencing, which produces massive amounts of DNA sequence fragments that researchers must assemble into a complete genome, in a process known as genome assembly. After the genome is assembled, genetic information must be decoded, in a process known as genome annotation. Genome assembly is a difficult task requiring a combination of a knowledge of computer science, mathematics, and molecular biology. During analysis of sequencing data from second-generation sequencing technologies, genome assembly requires a large amount of computing time and an enormous amount of computing memory. This demand for intensive computing resources has limited the assembly of many population-specific genomes or individual genomes.

To date, all biomedical research related to the human genome is based on the human reference genome [Genome Reference Consortium Human Build 38 (GRCh38)] produced by the HGP. Human genome annotation relies on databases such as RefSeq and GENCODE. Almost all biomedical human genome studies have been based on a “map-to-the reference-genome” strategy. However, the human reference genome produced by the HGP was sequenced from only several individuals; therefore, it does not reflect the overall genomic status of all populations^6^ and thus is incomplete. A recent publication has shown that 819 incoherent gaps remain in the human reference genome GRCh38, and some variations in long fragments cannot be matched to the existing reference genome sequence^7^. Consequently, researchers working on genomics have called for a more comprehensive human reference genome to be built, i.e., a human pan-genome sequence^8^.

## Pan-genome origin and status

The pan-genome refers to the entire genome composition of a clade, which can be a species, and encompasses the gene pool of all individuals in that clade (species). The pan-genome of a species consists of three parts: core genes, distributed genes, and individual-specific genes. Core genes are those found in all individuals of the species. In general, core genes are involved in the basic biological functions of the species and determine the main phenotype of the species. Distributed genes are those that exist in some individuals but are absent in others. An individual-specific gene is a gene that appears only in an individual of a species. The latter two types of genes are currently believed to be inessential for the basic life activity of a species; those genes are often involved in secondary metabolism or might increase the survival advantage of the species. The investigation of the pan-genome began as early as 2005 with a study of the *Streptococcus agalactiae* genome. In the case of *S. agalactiae*, each bacterial strain may have approximately 2,000 genes, but the complete genome of *S. agalactiae* can be integrated to approximately 6,000 genes^9,10^. Previous studies in lower organisms, such as bacteria, have suggested that if the number of sequenced individuals in a species increases, then the number of genes also increases, in which case the species is considered to have an open pan-genome. Otherwise, the species is thought to have a closed pan-genome, and after the number of sequenced individuals reaches a certain threshold, the gene numbers will not increase.

The first human pan-genome study was published internationally in 2010. That study analyzed the whole genome sequences of one Asian and one African individual, and then compared the differences between the sequences of the two individuals to the human reference genome. At least 19 Mbp–40 Mbp new sequences in the human genome were proposed to be absent in the human reference genome sequence^11^. The potential novel genes and biological functions of those newly discovered genomic sequences remain unclear. If more individual human genomes are genome-wide sequenced, the human genome reference sequence will be rewritten, thus providing insights into the importance of these novel sequences.

## HUPAN solves a technical bottleneck in pan-genome research

**Figure 1:**
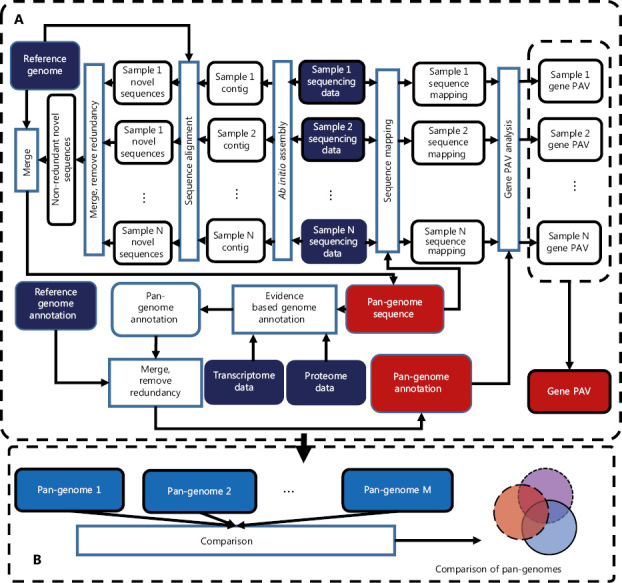
Schematic diagram of Human Pan-genome ANalysis (HUPAN). The system comprises two parts: the pan-genome construction pipeline (A) and pan-genome comparison analysis (B). In (A), by using the raw sequencing data of samples, *ab initio* genome assembly is first performed for each sample; the assembled contigs are then aligned to the reference genome to determine the novel sequences for each sample; these novel sequences for each samples are merged, and redundancy is removed by sequence comparison; the resultant non-redundant novel sequences are then merged with the reference genome to create the pan-genome; genome annotation is performed for the novel sequences of the pan-genome; the raw sequencing data of each sample are mapped to the pan-genome to determine the gene presence and absence variation (PAV) for each sample; and finally a PAV profile is created for the samples. In (B), different pan-genomes are compared to show the features of the target pan-genome.

With the development of second-generation sequencing technology and the decrease in sequencing costs, the number of whole genome sequencing of humans is rapidly increasing. Increased individual sequencing is a prerequisite for pan-genome research. Pan-genome research could provide scientists with opportunities to holistically understand the mysteries of human genetics and to explore the possible links between gene variation and certain diseases. However, the size of deep sequencing data of the human whole genome is enormous (at a sequencing depth of 30-fold, the raw sequencing data comprises approximately 90 Gb). The pan-genome research field expects to analyze a large number of individuals to discover the characteristics of a population; therefore, high-performance computer clusters with high memory and storage capacity are required for pan-genome study. Regarding software, no existing software for human pan-genome analysis is currently available, thus making choosing a proper program for pan-genome analysis difficult. After accessing the existing methods, we decided to develop a human pan-genome analysis tool through a collaboration among researchers from the faculties of Computer Science and Medicine of Shanghai Jiao Tong University. The new system, HUPAN, can construct a human pan-genome from all reads with drastically reduced memory requirements and improve computation efficiency. **Figure 1** shows a diagram of the HUPAN system. First, the raw data from whole genome deep sequencing were assembled with a memory-efficient program called SGA, and the assembled new sequences that did not match the human reference genome published by the HGP were extracted for each individual. The newly extracted sequences were then divided into completely unaligned sequences and partially unaligned sequences according to the alignment of contigs to the human reference genome. These novel sequences were then combined completely unaligned and partially unaligned sequences, and redundancy was removed for these two types of novel sequences. New protein-coding genes were predicted for the completely unaligned new sequences. The predicted new genes were validated at the genomic and transcriptional levels. The new genes combined with the human reference genome composed a human pan-genome. After the human pan-genome was built, the raw sequencing data for each individual were mapped directly to the pan-genome reference sequence, and the presence and absence variation (PAV) of genes could then be determined for each individual.

Most existing pan-genome methods use only unaligned reads. However, our results showed that using unmapped/unaligned reads alone generates more misassembled sequences, thereby introducing more false predictions of novel genome sequences in pan-genome analysis. Using all reads instead of the unmapped/unaligned reads alone can significantly improve pan-genome quality. HUPAN is the first program to use all sequencing reads for each individual in a large-scale human pan-genome analysis. By using all sequencing reads, HUPAN achieves lower mis-assembly rates. HUPAN has been granted a computer software copyright in China (2019SR0058204). The cost of developing a better pan-genome is primarily due to the requirement for a much larger computational resource consumption. Our test results showed that whole genome sequencing data assembly for one individual requires approximately 60 Gb of memory and 70 hours of computation time. With a very large number of samples (such as a few thousand or more), we recommend using the unmapped/unaligned reads alone instead of all reads for pan-genome analysis. For a few hundred genomes or fewer, HUPAN is a better option for pan-genome analysis.

Using HUPAN, we successfully analyzed whole genome sequencing data from an additional 90 Han Chinese individuals and 910 individuals of African descent, and compared these pan-genomes with the pan-genome derived from whole genome sequencing data from human stomach mucosa. The Han Chinese pan-genome was constructed with all genetic sequences from the 275 (185 + 90) Han Chinese individuals. We also proposed a unique gene set for Han Chinese^1^. For instance, the pan-genome derived from 275 Han Chinese individuals showed 29.5 Mbp novel sequences and 188 novel protein coding genes absent in the human reference genome. PAV analysis of whole genome deep sequencing data for 185 Han Chinese individuals revealed 606 distributed genes, 116 of which were novel and 490 of which were in the human reference genome. Function enrichment analysis indicated that the distributed genes were enriched in defense/immune responses.

## Impact of HUPAN on future cancer research

After many individuals are sequenced, the sequencing data of a large-sized population can be used to build a pan-genome. Then, the pan-genome can serve as a reference genome to study the discrepancies between an individual genome and the pan-genome. Thus, a pan-genome study can reveal that everyone has not the same gene set. Studying the differences in gene PAVs among individuals and further exploring the associations of certain diseases or tumors with PAVs may be a future research direction.

As human whole genome sequencing continues to grow, scientists can build a human pan-genome reference sequence. Many people are concerned about questions such as “How many genes do I have; what are the chances that the distributed genes I have are related to susceptibility to various diseases or cancers; and can genes that make me susceptible to a disease or a cancer be treated through scientific strategies?” Advances in the field may allow such questions to be addressed in the future.

Researchers in biomedicine may use HUPAN to study different diseases and different cancers in detail. They would be able to determine the number of differences between individual genomes and whether these differences are related to certain diseases or cancer susceptibility. In particular, special attention should be paid to some important genes associated with disease susceptibility across diverse populations or geographies. PAV is likely to reveal important genes that are responsive or resistant to certain therapeutic drugs. HUPAN is expected to play an important role in pan-genome research on diseases and tumors. The genes that cause susceptibility to certain diseases are highly unlikely to have already been found. In pan-genomics research, the analytic route for genes is different from that in traditional human genome analysis. For core gene analysis, researchers can focus on single-nucleotide polymorphisms (SNPs) and insertion/deletion (InDel) variations, whereas for distributed gene analysis and individual-specific genetic analysis, more attention should be paid to PAV.

In summary, people hope to discover their own genomes as comprehensively as possible. When the human pan-genome reference sequence is finally successfully constructed, the research model for biomedicine for human genomes, especially for cancer genomes, will change from the “map-to-the reference genome” strategy to the “map-to-the reference pan-genome” strategy. The human pan-genome will provide more comprehensive genetic information than the existing human reference genome.

## References

[1] Duan Z, Qiao Y, Lu J, Lu H, Zhang W, Yan F (2019). HUPAN: a pan-genome analysis pipeline for human genomes. Genome Biol.

[2] Francis S.,  Collins FS, Morgan M, Patrinos A (1990). The human genome project: lessons from large-scale biology. Science.

[3] Li YQ (1993). China launches genome project. Nature.

[4] Pennisi E (2003). Reaching their goal early, sequencing labs celebrate. Science.

[5] Goodwin S, McPherson JD, McCombie WR (2016). Coming of age: ten years of next-generation sequencing technologies. Nat Rev Genet.

[6] Schneider VA, Graves-Lindsay T, Howe K, Bouk N, Chen H-C, Kitts PA (2017). Evaluation of GRCh38 and *de novo* haploid genome assemblies demonstrates the enduring quality of the reference assembly. Genome Res.

[7] Audano PA, Sulovari A, Graves-Lindsay TA, Cantsilieris S, Sorensen M, Welch AE (2019). Characterizing the major structural variant alleles of the human genome. Cell.

[8] Yang X, Lee WP, Ye K, Lee C (2019). One reference genome is not enough. Genome Biol.

[9] Tettelin H, Masignani V, Cieslewicz MJ, Donati C, Medini D, Ward NL (2005). Genome analysis of multiple pathogenic isolates of *Streptococcus agalactiae*: Implications for the microbial ‘pan-genome’. Proc Natl Acad. Sci. USA.

[10] Lefébure T, Stanhope MJ (2007). Evolution of the core and pan-genome of Streptococcus: positive selection, recombination, and genome composition. Genome Biol.

[11] Li R, Li Y, Zheng H, Luo R, Zhu H, Li Q (2010). Building the sequence map of the human pan-genome. Nat Biotech.

